# Acute Acquired Comitant Esotropia in Adults: Is It Neurologic or Not?

**DOI:** 10.1155/2016/2856128

**Published:** 2016-11-27

**Authors:** Kadriye Erkan Turan, Tulay Kansu

**Affiliations:** ^1^Department of Ophthalmology, Faculty of Medicine, Hacettepe University, Ankara, Turkey; ^2^Department of Neurology, Faculty of Medicine, Hacettepe University, Ankara, Turkey

## Abstract

*Objectives*. Acute acquired comitant esotropia (AACE) can be a diagnostic challenge for ophthalmologists and neurologists because of its association with neurological pathologies. Our study describes a series of adult patients with AACE of undetermined etiology.* Methods*. Data on the clinical findings of patients presented with AACE of undetermined etiology with a minimum follow-up of 1 year were retrieved from the medical records and the results analyzed.* Results*. A series of 9 esotropia cases (age range: 20–43 years) was reviewed. All patients had full duction and versions, without an A-pattern or V-pattern. All patients had esotropia for distance and near. Neurological evaluation in all cases was normal. Among patients, 3 were treated with prisms, 4 were treated with strabismus surgery, and 1 was treated with botulinum toxin injections; 1 patient declined treatment. In treated patients posttreatment sensory testing indicated restoration of binocularity that remained stable throughout follow-up of 1–9 years. The patient that declined treatment had binocular function with base-out prisms.* Conclusion*. Acute onset esotropia may be seen without a neurological pathology in adults. Good motor and sensory outcomes can be achieved in these patients with AACE of undetermined etiology via surgical and nonsurgical methods.

## 1. Introduction

Acute acquired comitant esotropia (AACE) is an unusual presentation of esotropia that occurs in older children and adults. AACE is characterized by acute onset of a relatively large angle of esotropia, along with diplopia and minimal refractive error [1, 2]. AACE is not cyclical, although it may initially be intermittent. It is comitant at distance and near fixation [1]. AACE is categorized as 3 types, based on the clinical features and apparent etiology: type 1 (Swan type): acute onset esotropia following occlusion; type 2 (Franceschetti type): refractive error which is minimal hypermetropia without an accommodative element; type 3 (Bielschowsky type): AACE associated with myopia [3, 4]. The other causes of acute esotropia in adults include sixth nerve palsy, age-related distance esotropia, divergence palsy, accommodative esotropia, decompensated monofixation syndrome, restrictive strabismus, consecutive esotropia, sensory strabismus, ocular myasthenia gravis, and some neurological disorders (tumors of the cerebellum, brainstem, pituitary region, corpus callosum, Arnold-Chiari malformation, cerebellar disease, and idiopathic intracranial hypertension). AACE is considered rare, but no statistical data is available regarding its actual incidence or prevalence [5]. We present 9 patients with AACE of undetermined etiology and a review of the relevant literature.

## 2. Methods

The medical records of 9 consecutive patients older than 18 years of age presenting with acute acquired comitant esotropia of undetermined etiology between 1993 and 2014 and with a minimum follow-up of 1 year were reviewed retrospectively. The authors adhered to the tenets of the Declaration of Helsinki. All patients underwent ophthalmological, neurologic examinations and orbital-cranial MRI with contrast for inclusion. An ophthalmologist and neurologist performed the examination. Patients with incomitant esotropia were excluded. Incomitance was defined as limited abduction and larger deviation at lateral gaze.

The following information was obtained on each patient: sex, age, presenting complaint, duration of symptoms, signs, cycloplegic refraction with cyclopentolate, best corrected visual acuity, deviation at near and distance, fusion and stereopsis, neurological examination and tests, cranial and orbital MRI findings, treatment, follow-up time, and outcomes. Snellen chart was used to test visual acuity. Sensory fusion was evaluated with Worth 4-dot test at near (1/3 m) and distance (6 m) and stereopsis was assessed with Titmus test. The angles of deviations were assessed by alternate cover (prism and cover) test in all 9 cardinal gaze positions. Both near and distance measurements were taken. Ocular motility, patterns, and nystagmus were evaluated clinically. Lees screen test was performed.

## 3. Results

The 9 patients presented with acute onset of binocular horizontal diplopia that developed 10 days–18 months prior to presentation. Diplopia was constant throughout the day in all cases. Case details are given in Table 1.

The 5 female and 4 male patients were aged 20–43 years. None of the patients had a history of recent trauma, occlusion of one eye, or recent illness. The medical history in all cases was unremarkable. Best corrected visual acuity was 1.0 in all eyes. All patients had cycloplegic spherical equivalent refraction between −1.50 and +0.50 diopters. All patients correctly identified the Ishihara color plates with each eye. External and anterior segment examinations and fundoscopic examinations in all patients were normal. All the patients had full duction and versions and no A-pattern or V-pattern. These patients were initially suspected to have lateral rectus paresis, but our examination showed no slowing of abducting saccades and full abduction assessed clinically. Lees screen tests of patients showed no limitation of abduction. None of the patients had nystagmus. In each patient esodeviation was comitant and there was no difference in the angle of strabismus with either eye fixating. All patients had equal amounts of esotropia with a range from 16 to 45 prism diopters for distance and near fixation.

In addition to ophthalmic examination, each patient was examined by a neurologist and underwent an MRI with contrast. Neurologic evaluation in all cases was normal. Myasthenia work-up with Tensilon test in 4, single fiber EMG in 5, and acetylcholine receptor antibody in 3 patients were negative (Table 1). In all, 8 of the patients had normal cranial MRI findings, whereas 1 patient had a simple pineal cyst. Eight patients had coexisting medical diseases.

**Figure 1:**
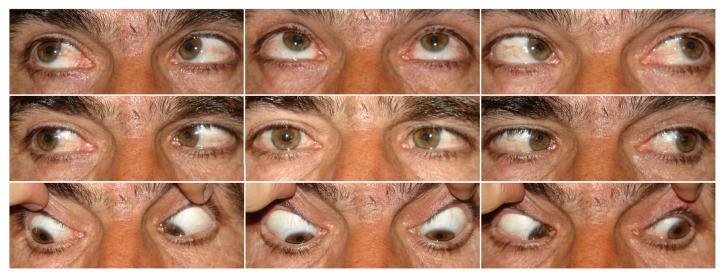
Nine cardinal photographs of patient number 3 showing left esotropia with full versions.

**Figure 2:**
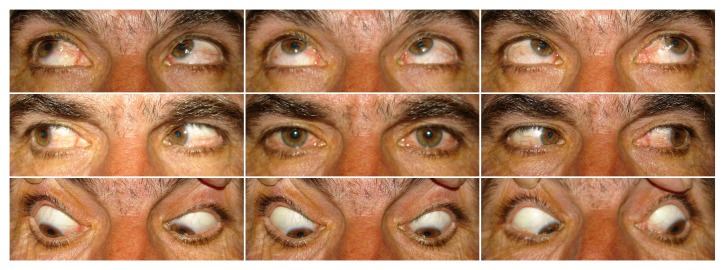
Nine cardinal photographs of patient number 3 after botulinum toxin injection.

In total, 8 patients were treated successfully (3 with prisms, 4 with strabismus surgery, and 1 with botulinum toxin injections), whereas 1 patient (patient number 4) declined treatment. Patients 1, 5, 7, and 9 underwent recession/resection surgery, and all regained binocularity on Worth 4-dot test at near and distance. In patients 2, 6, and 8 binocular fusion on Worth 4-dot test was restored with prism therapy. Botulinum toxin A injections were administered to patient 3, who did not want to undergo surgical correction (Figure 1); the patient required 3 injections and fused afterwards on Worth 4-dot test (Figure 2).

In all 8 of the treated patients posttreatment sensory testing indicated restoration of binocularity; all 8 patients regained stereopsis of 100 sec of arc that remained stable throughout 1–9 years of follow-up. Patient 4, who declined treatment, had binocular fusion with base-out prisms at near on Worth 4-dot test.

## 4. Discussion

AACE is classified as 3 types. Common to all 3 types is acute onset, concomitancy, a relatively large angle of deviation, good binocular potential, and no underlying neurological disease [5]. Type 1 AACE (Swan type) follows occlusion or loss of vision in one eye secondary to injury or disease [23]. Type 2 AACE (Franceschetti type) is characterized by acute onset of a relatively large angle of comitant esotropia and diplopia [3]. The refractive error is usually a minimal degree of hypermetropia and no accommodative element is detected [5]. The pathogenesis of type 2 AACE remains to be fully elucidated. Surgery was required to reestablish ocular alignment in all patients described to date [5]. The patients in this series represent this group. Type 3 AACE (Bielschowsky type) is characterized by acute onset of esotropia in patients with uncorrected myopia of −5.00 diopters or more, presumably following physical or mental stress [24]; subsequent reports have emphasized that good binocular function can be maintained in these patients with prisms [5].

Tumors of the cerebellum, brainstem, pituitary region, and corpus callosum can be associated with acute onset esotropia [5]. Anderson and Lubow [6] reported a patient with acute onset esotropia that had an astrocytoma of the corpus callosum; esotropia spontaneously resolved before surgery and radiotherapy. Zweifach [7] reported a 10-year-old boy that developed esodeviation and had negative neurological and neuroradiological findings. Successful surgical repair was performed 10 months after the onset of strabismus. Clinical signs that resulted in the diagnosis of a brain tumor (medulloblastoma) were observed 18 months after surgery (28 months after the onset of diplopia). This case shows that periodic neurological reevaluation is necessary in cases of acquired strabismus, even following successful surgical correction [7]. Williams and Hoyt [8] described 6 children with tumors of the brain stem or cerebellum and AACE. None of the 6 patients developed signs of abducens nerve dysfunction. Liu et al. [25] reported 30 children with esodeviation due to neurological insult. Among the comitant esotropia patients (12 patients), 10 had brain tumors, one had meningitis, and one had a basilar artery aneurysm and associated thrombosis.

Arnold-Chiari malformation has been associated with esotropia in several cases [11, 13]. Akman et al. [9] reported 2 patients with craniocervical junction anomaly. They posited that divergence palsy due to brainstem dysfunction was the etiological mechanism of esodeviation in their cases. The association between acute onset concomitant esotropia in patients with Arnold-Chiari syndrome has also been attributed to coexisting hydrocephalus [5]. Acute comitant strabismus is also well documented in patients with intracranial tumor and no hydrocephalus [22]. Patients with mild sixth nerve palsy can recover quickly, but a comitant, moderate angled esotropia may remain [26]. Jampolsky [27] noted that the small V-pattern observed when deviation is still concomitant should indicate the possibility that the primary problem is paresis of the abducens nerve [5]. Idiopathic intracranial hypertension is also an occasional cause of comitant esotropia [16].

Wong et al. [18] presented 7 adult patients with cerebellar ataxia. They suggested that cerebellar esotropia might also be caused by excessive convergence tone, a supranuclear phenomenon that might result from disruption to the central vestibular system. The presence of abducting nystagmus is obviously an important feature in patients with AACE when investigating for subtle sixth nerve involvement [4]. Abducting nystagmus has been noted in patients diagnosed with central nervous system pathology [4, 5].

AACE can occur in patients without any neurological pathology. Rather than the three types of comitant deviation, decompensation of preexisting phoria or monofixation syndrome has been reported in 9 patients, as a common cause of this presentation [22]. Lyons et al. [22] emphasized that a high index of clinical suspicion should be maintained in the absence of expected findings associated with acute comitant esotropia, such as hypermetropia, fusion potential, atypical features, and neurologic signs. Malbran and Norbis [19] described 4 siblings that developed acute comitant esotropia at ages 6–9 years. Although 1 patient had a partially accommodative component, all 4 ultimately required surgery. Burian and Miller [3] reported 8 patients aged 6.5–72 years, of which one had a preceding illness. None of the patients had a neurological condition, but all had diplopia and underwent surgery. Goldman and Nelson [1] reported 2 children aged 5 and 7 years that had acute onset of diplopia, a relatively large angle of comitant esotropia, and minimal hyperopic refractive error of unknown etiology. Clark et al. [2] described 6 children aged 5–11 years that developed acute nonaccommodative esotropia with diplopia. In 3 of the patients esotropia and diplopia were initially intermittent but became persistent within a few weeks. All of the patients had normal neurological findings. The researchers posited that each case was an unusual presentation of esotropia of undetermined etiology. This type of acute onset comitant esotropia was also described in monozygous twins [20]. Excessive smartphone usage is also reported as a cause of AACE in adolescents [28]. Neurological disorders associated with esotropia are listed in Table 2, and some of previously reported cases of AACE with unknown etiology including both children and adults are shown in Table 3.

Divergence insufficiency (DI) or weakness refers to esotropia at distance fixation only in the setting of normal lateral rectus muscle function, which differentiates it from abducens palsy [29]. DI has been described in all age groups, and individuals with isolated DI usually do not have a serious neurological condition [30, 31]. Divergence palsy is acute onset distance esotropia with divergence loss in all age groups and is typically associated with various neurological diseases, such as brain tumors, multiple sclerosis, trauma, subdural hematoma, cerebrovascular disease, head trauma, and tertiary syphilis [32–34]. Abducens palsy is a common cause of esotropia that is more severe at distance than at near fixation. Complete palsy of one or both abducens nerves is characterized by large esodeviation in primary gaze and clinically marked limitation of abduction, with lateral incomitance in unilateral or asymmetric cases. Incomplete or partial abducens palsy is characterized by a smaller esodeviation in primary gaze with variable limitation of abduction and smaller amounts of incomitance in lateral gaze [35]. The manifestations of DI, divergence palsy, and mild bilateral sixth nerve palsy can be quite similar, making clinical differentiation difficult [30].

*Causes of Esotropia in Adults*

Paralytic strabismus
  - Sixth nerve palsy
Age-related distance esotropia
Sagging eye syndrome
Acute acquired comitant esotropia
  - Swan type, occlusion related
  - Franceschetti type, idiopathic
  - Bielschowsky type, associated with myopia
Divergence insufficiency
Divergence palsy
Accommodative esotropia
Decompensated monofixation syndrome
Restrictive strabismus
Sensory strabismus
Consecutive esotropia
Thyroid ophthalmopathy
Ocular myasthenia gravis
Chronic progressive external ophthalmoplegia
Heavy eye syndrome

Strabismus in high myopes is described as “Heavy Eye” syndrome (myopic strabismus fixus) in which there is inferior displacement of the lateral rectus (LR) that results in esotropia and hypotropia with limited supraduction and abduction. It is related to a possible mechanical contact between the enlarged globe and the lateral orbital wall in high myopes [36]. Similar clinical and radiographic features are also seen in elderly nonmyopic patients first described in 2009 as Sagging Eye Syndrome (SES). It is a cause of acquired, small-angle horizontal and vertical strabismus that most often occurs in elderly patients [36]. The strabismus is secondary to involutional changes in the extraocular muscles and orbital connective tissues, which result in inferomedial displacement of the lateral rectus muscle [37]. Our patients were young adults (age 20–43 years) and they did not have blepharoptosis, high upper lid creases, and deep superior sulci suggesting SES as the diagnosis.

There is an overlap in understanding of SES and age-related distance esotropia (ARDET). ARDET is an acquired small comitant esodeviation at distance fixation. Horizontal diplopia only at distance fixation may be intermittent or persistent. This form of esotropia is observed in elderly patients and is not associated with LR muscle underaction or with any known neurological pathology [29, 38, 39]. ARDET is considered a form of divergence insufficiency, although some consider it a distinct clinical entity [29, 38, 40]. ARDET shares some features with AACE types 2 and 3 but differs from them in several ways. Type 2 (Franceschetti) AACE affects individuals of all ages, and patients usually have esotropia at both distance and near fixation, and type 3 (Bielschowsky) AACE is associated with myopia [29]. Patients with ARDET can experience a slight increase in distance esodeviation over time and a slow decrease in fusional divergence amplitudes with normal ocular motility [39]. Sagging and downward displacement of the muscle pulleys is thought to be a cause of ARDET. New-onset ocular misalignment in adults is strongly associated with age and the highest incidence is among individuals in the eighth decade of life [41].

Some researchers suggest that comitant esotropia, in contrast to incomitant esodeviation, is benign and does not warrant further neurological investigation [5]. However, concomitancy in acute onset esotropia does not rule out the possibility of an underlying serious neurological condition [8]. It was reported that comitant strabismus might result from involvement of supranuclear mesencephalic structures that control vergence eye movement whereas others posited that is was due to infranuclear pathologies [5, 42].

It is recommended that patients who present with acute onset diplopia and comitant esotropia should undergo complete ophthalmological and neurological examinations, and thorough anamnesis must be obtained to rule out cyclic esotropia, divergence insufficiency, paretic strabismus, and myasthenia gravis [2]. A high index of clinical suspicion should be maintained and neuroimaging should be considered in the absence of expected findings associated with acute comitant esotropia, such as hypermetropia, fusion potential, and atypical features [22]. It is suggested that neuroradiological imaging should be performed in patients without hyperopic refraction or still have esotropia with full hyperopic prescription [26]. In older patients with a clear history of acquired esotropia it is suggested that prompt neuroimaging be performed, regardless of the presence of other neurological signs or symptoms. In patients with acute onset comitant esotropia that cannot demonstrate motor fusion when examined with prisms or a synoptophore the possibility of an underlying neurological disease should be suspected [5].

The primary aim of strabismus management in patients with AACE is restoration of normal ocular alignment and a reduction in diplopia, if present, which helps patients perform daily activities, normally. There are various treatment options for patients with AACE, including both surgical and nonsurgical approaches. Nonsurgical treatments include ocular occlusion, prism therapy, and botulinum toxin type A injection [43]. Prism correction of diplopia is a valuable nonsurgical treatment option that can be especially beneficial in patients with small-angle strabismus (less than 15 prism diopters).

The patients in the present study are good examples of type 2 AACE, because none had apparent neurological problems and their esotropia did not resolve with correction of refractive error. However, the patients presented with symptoms 10 days to 18 months prior to presentation. Although the patients remarked any change in their symptoms, the authors cannot fully eliminate the possible presence of a prior sixth nerve paresis that has partially recovered by the time the patients presented. All 9 of the presented patients had stereoacuity, which eliminated the possibility of a previous monofixation syndrome that decompensated. Binocularity was restored with prism therapy, strabismus surgery, and botulinum toxin injections in all of the presented patients.

## 5. Conclusion

Our findings support the benign nature of AACE. We agree that the adults who develop acute esotropia must undergo careful motility analysis to rule out a paretic deviation and neuroimaging should be performed regardless of the presence of other neurological signs or symptoms. Cycloplegic refraction should be done to rule out the accommodative component especially in young patients. Abducting nystagmus and lack of binocularity may be signs of an underlying neurological disease. AACE patients without neurological disease can achieve good motor and sensory outcomes with appropriate treatment.

## Figures and Tables

**Table 1:** Clinical characteristics of patients with acute acquired comitant esotropia.

Patient number	Sex	Age (years)	Duration of symptoms	Presenting complaint	Sign	Vision		Deviation (PD)		Neurological examination	Investigations	Treatment	Surgical intervention	Prism glasses	Follow-up (years)	Outcome
						RE	LE	D	N							
1	M	32	6 months	Diplopia	Right esotropia	1.0	1.0	40	40	Normal	Cranial and orbital MRI: normal Tensilon test: negative	Surgery	RE MR Rc 5.5 mm, RE LR Re 6.5 mm	—	8	No manifest deviation

2	M	29	10 months	Diplopia	Right esotropia	1.0	1.0	18	16	Normal	Cranial and orbital MRI: normal Tensilon test: negative	Prism	—	16 PD BO Ground-in Both eyes	9	No manifest deviation with prism

3	M	43	12 months	Diplopia	Left esotropia	1.0	1.0	35	40	Normal	Cranial and orbital MRI: normal Single fiber EMG: negative Acetylcholine receptor ab: negative	Botulinum toxin injections	—	—	2	No manifest deviation after botulinum toxin injections

4	M	23	4 months	Diplopia	Right esotropia	1.0	1.0	45	45	Normal	Cranial and orbital MRI: normal Single fiber EMG: negative Acetylcholine receptor ab: negative	Refused any treatment	—	—	2	No change

5	F	43	18 months	Diplopia	Left esotropia	1.0	1.0	30	25	Normal	Cranial and orbital MRI: normal Single fiber EMG: negative	Surgery	LE MR Rc 5.0 mm, LE LR Re 5.5 mm	—	6	Intermittent 6 pd left esotropia

6	F	20	15 days	Diplopia Headache	Right esotropia	1.0	1.0	20	20	Normal	Cranial and orbital MRI: simple pineal cyst Cranial MR angiography: normal Single fiber EMG: negative	Prism	—	20 PD BO Ground-in Both eyes	4	No manifest deviation with prism

7	F	22	1 months	Diplopia	Left esotropia	1.0	1.0	25	25	Normal	Cranial and orbital MRI: normal Tensilon test: negative	Surgery	LE MR Rc 4.0 mm, LE LR Re 5.5 mm	—	3	No manifest deviation

8	F	32	6 months	Diplopia	Left esotropia	1.0	1.0	16	18	Normal	Cranial and orbital MRI: normal Tensilon test: negative Acetylcholine receptor ab: negative	Prism	—	16 PD BO Ground-in Both eyes	2	No manifest deviation with prism

9	F	27	10 days	Diplopia	Left esotropia	1.0	1.0	40	35	Normal	Cranial and orbital MRI: normal Single fiber EMG: negative	Surgery	LE MR Rc 5.5 mm, LE LR Re 6.5 mm	—	1	No manifest deviation

F: female, M: male, RE: right eye, LE: left eye, PD: prism diopters, D: distance, N: near, MRI: magnetic resonance imaging, EMG: electromyography, ab: antibody, MR: medial rectus, LR: lateral rectus, Rc: recession, Re: resection, and BO: base-out.

**Table 2:** Acute acquired comitant esotropia associated with neurological pathologies.

Author	Number of patients	Age (years)	Diagnosis	Treatment	Outcome/comment
Anderson and Lubow [6] (1970)	1	6	Astrocytoma of corpus callosum	Spontaneously resolved	(i) Papilledema (ii) Hemiplegia

Zweifach [7] (1981)	1	10	Medulloblastoma	Strabismus surgery	(i) Failure of reestablishing binocularity

Williams and Hoyt [8] (1989)	6		Tumors of brain stem or cerebellum	Strabismus surgery Neurosurgical treatment	(i) Nystagmus (ii) Failure of reestablishing binocularity

Akman et al. [9] (1995)	2	13–35	Arnold-Chiari malformation Basilar impression	Posterior fossa decompression (1 patient)	(i) No difference

Simon et al. [10] (1996)	1	5	Cerebellar astrocytoma	Strabismus surgery	(i) Restored BSV

Lewis et al. [11] (1996)	5	17–36	Chiari 1 malformation	Posterior fossa decompression (4 patients)	(i) Gaze-evoked nystagmus (ii) Restored BSV (4 patients)

Dikici et al. [12] (1999)	1	5	Cerebellar astrocytoma	Neurosurgical treatment	(i) Without diplopia (ii) Papilledema

Biousse et al. [13] (2000)	4	5–37	Chiari 1 malformation	Suboccipital decompression Strabismus surgery	(i) Restored BSV

Defoort-Dhellemmes et al. [14] (2002)	1	9	Chiari 1 malformation	Suboccipital decompression	(i) Restored BSV

Hentschel et al. [15] (2005)	1	5	Chiari 1 malformation	Posterior fossa decompression	(i) Recovery of binocular fusion

Parentin et al. [16] (2009)	1	9	Idiopathic intracranial hypertension	Lumbar puncture Medical therapy	(i) Orthophoria

Kemmanu et al. [17] (2012)	2	5–7	Pontine glioma	Neurosurgical treatment	(i) Nystagmus (ii) Papilledema

Wong et al. [18] (2015)	7	31–75	Cerebellar ataxia	Prism Botulinum toxin injections Strabismus surgery	(i) Progression over time (ii) Restored BSV

BSV: binocular single vision; PD: prism diopters.

**Table 3:** Acute acquired comitant esotropia with unknown etiology.

Author	Number of patients	Age (years)	Diagnosis	Treatment	Outcome/comment
Malbran and Norbis [19] (1956)	4	6–9	Undetermined etiology	Strabismus surgery	(i) Siblings

Burian and Miller [3] (1958)	8	6–72	Undetermined etiology	Strabismus surgery	(i) Binocular potential

Goldman and Nelson [1] (1985)	2	5–7	Undetermined etiology	Strabismus surgery	(i) Esotropia of 8 PD (ii) Orthophoria

Clark et al. [2] (1989)	6	5–11	Undetermined etiology	Strabismus surgery	(i) Orthophoria (ii) One recurrent esotropia

Ahmed and Young [20] (1993)	2	4-5	Undetermined etiology	Strabismus surgery	(i) Twins (ii) Restored BSV

Simon and Borchert [21] (1997)	10	5–35	1/10 had neurological disease 7/10 had refractive element	Optical correction Strabismus surgery	(i) Esotropia improved

Lyons et al. [22] (1999)	10	3.5–24	1/10 had cerebellar astrocytoma 9/10 had hypermetropia	Optical correction Strabismus surgery	(i) Uncorrected hypermetropia (ii) Decompensated monofixation syndrome

BSV: binocular single vision; PD: prism diopters.
